# Attenuated Neural Processing of Risk in Young Adults at Risk for Stimulant Dependence

**DOI:** 10.1371/journal.pone.0127010

**Published:** 2015-06-15

**Authors:** Martina Reske, Jennifer L. Stewart, Taru M. Flagan, Martin P. Paulus

**Affiliations:** 1 Department of Psychiatry, University of California San Diego, La Jolla, California, United States of America; 2 Institute of Neuroscience and Medicine (INM-6), Computational and Systems Neuroscience and Institute for Advanced Simulation (IAS-6), Theoretical Neuroscience, Forschungszentrum Jülich GmbH, Jülich, Germany; 3 JARA BRAIN Institute I, Jülich, Germany; 4 CUNY Queens College, Queens, New York, United States of America; 5 University of Texas, Austin, Texas, United States of America; 6 Psychiatry Service, VA San Diego Healthcare System, La Jolla, California, United States of America; 7 Laureate Institute for Brain Research, 6655 S Yale Ave, Tulsa, Oklahoma, United States of America

## Abstract

**Objective:**

Approximately 10% of young adults report non-medical use of stimulants (cocaine, amphetamine, methylphenidate), which puts them at risk for the development of dependence. This fMRI study investigates whether subjects at early stages of stimulant use show altered decision making processing.

**Methods:**

158 occasional stimulants users (OSU) and 50 comparison subjects (CS) performed a “risky gains” decision making task during which they could select safe options (cash in 20 cents) or gamble them for double or nothing in two consecutive gambles (win or lose 40 or 80 cents, “risky decisions”). The primary analysis focused on risky versus safe decisions. Three secondary analyses were conducted: First, a robust regression examined the effect of lifetime exposure to stimulants and marijuana; second, subgroups of OSU with >1000 (n = 42), or <50 lifetime marijuana uses (n = 32), were compared to CS with <50 lifetime uses (n = 46) to examine potential marijuana effects; third, brain activation associated with behavioral adjustment following monetary losses was probed.

**Results:**

There were no behavioral differences between groups. OSU showed attenuated activation across risky and safe decisions in prefrontal cortex, insula, and dorsal striatum, exhibited lower anterior cingulate cortex (ACC) and dorsal striatum activation for risky decisions and greater inferior frontal gyrus activation for safe decisions. Those OSU with relatively more stimulant use showed greater dorsal ACC and posterior insula attenuation. In comparison, greater lifetime marijuana use was associated with less neural differentiation between risky and safe decisions. OSU who chose more safe responses after losses exhibited similarities with CS relative to those preferring risky options.

**Discussion:**

Individuals at risk for the development of stimulant use disorders presented less differentiated neural processing of risky and safe options. Specifically, OSU show attenuated brain response in regions critical for performance monitoring, reward processing and interoceptive awareness. Marijuana had additive effects by diminishing neural risk differentiation.

## Introduction

The use of cocaine, prescription amphetamines and methylphenidate (which are referred to as “stimulants”) for non-medical purposes by young adults to enhance performance in academic and/or social situations poses an increasing public health problem [1]. For example, up to 16% of individuals experimenting with cocaine develop dependence within 10 years [2]. Exposure to drugs of abuse particularly during brain maturation in adolescence and young adulthood increases the risk of future dependence [3]. Exposed to highly competitive social and academic situations, 7–8% of U.S. [4, 5] and 20% of German [6] college students report the off-prescription use of cognitive enhancers, for example of stimulants, in the past year. Up to 17% of male and 11% of female undergraduates admit lifetime use of prescription stimulants [6, 7].

Despite this growing trend, few studies have examined biological and behavioral processes that might distinguish individuals who use these drugs versus those who do not, providing insights into precursors of stimulant dependence. In particular, previous research typically compared stimulant dependent individuals with non-using healthy subjects. Such study designs, however, make it impossible to differentiate a predisposing vulnerability reflected in behavioral performance or neural activation patterns from effects of repeated exposure to neurotoxic substances and may have misattributed results as a consequence of cumulative use. Longitudinal study designs starting with adolescents prior to any substance use would be ideal, but these require an enormous effort and resources. Characterizing phenotypes indicative of a heightened risk for stimulant dependence by investigating users at very early stages of use on the other hand allows for the identification of potential precursors of substance related problems. Therefore, the present study investigates brain activation and behavioral performance in a substantive sample of young occasional stimulant use (OSU) with very limited use. The identification in brain and behavioral differences in such OSU could suggest that these presumably preceded the use of stimulants as cumulative neurotoxic exposure is limited.

As a growing literature implicates impaired decision making in stimulant dependent individuals in high-risk situations [8], this investigation examined whether deficits in decision making extend to OSU. Risk taking involves selecting an action associated with an uncertain payoff, the possibility of a beneficial outcome at the potential cost of an adverse outcome [9–11] wherein individuals need to establish a balance between obtaining rewards and avoiding losses [12–14]. An important variant is decision making under ambiguity, where outcomes are unknown or have unknown probability distributions (see e.g. [15]). Learning from outcomes, particularly from punishment, is pivotal and relies on the ability to respond to changing contingencies by inhibiting a dominant response, and monitoring responses and outcomes. Research shows that individuals with cocaine abuse or dependence exhibit similar risky behavior in presence of high or low penalties, whereas healthy individuals choose less risky options in the face of high punishment [16]. Moreover, individuals with cocaine, methamphetamine or marijuana abuse and/or dependence choose greater numbers of risky options than comparison subjects, particularly on tasks requiring the evaluation of the magnitude and timing of rewards and punishments [17–22]. Although prior work demonstrates that, like stimulant dependent individuals, OSU exhibit increased risk-taking behavior but successful behavioral adjustment after punishment [23], no studies have simultaneously examined brain and behavior performance during risky decision making in a large sample of OSU. Hence, the present study examined neural and behavioral indices in OSU and stimulant naïve comparison subjects (CS) during a “risky gains” decision making paradigm [23–25].

Main foci for imaging analyses were prefrontal cortices including dorsolateral prefrontal cortex (DLPFC), superior (SFG) and inferior frontal gyri (IFG), medial frontal cortex (including the anterior cingulate cortex, ACC) as well as insula and caudate. In CS, prefrontal recruitment during voluntary risk taking is thought to reflect maintaining task objectives in short term memory, discerning reward value of available choices, and avoidance of punishment by inhibiting risky decisions [26, 27], while risk-related involvement of insula and ACC have been associated with calculation of risk prediction error, as well as the difference between expected versus actual risk computed when reward probabilities are uncertain [24, 28–31]. Specifically, dorsal ACC (dACC) activation relates to tonic aspects of cognitive control and goal-oriented behavior during decision making. It raises with increasing risk and recruits prefrontal brain regions to avoid further risk [32]. Subgenual (sgACC) and rostral ACC (rACC) play essential roles in hedonic evaluations and motivational aspects of decision making [32–34]. The caudate, a component of the dorsal striatum, has been linked to motivational and learning-related aspects of goal-directed actions [35]. Its role in decision making is based on dopamine-mediated reinforcement processes and comprises choice-outcome contingency learning via feedback, a mechanism reinforcing behaviors with positive outcomes and avoiding choices associated with losses, linking risky decisions with positive and negative outcomes. Lastly, insula activation during decision making has been conceptualized to reflect a bodily representation of risk as interoceptive signals reach the posterior insula to travel to the anterior insula to generate interoceptive awareness [36, 37].

Given that research in stimulant dependent subjects has indicated hypoactivations compared to non-using comparison subjects in these brain areas [19, 38–47] the present study sought to determine whether neural activation patterns described in stimulant dependent individuals extend to OSU. Following primary analyses focusing on overall and risk-specific behavioral and neural decision making differences between OSU and CS, three secondary analyses were carried out. First, we investigated whether cumulative stimulant use moderated results in this cross-sectional study within OSU. Respective findings will explicate whether differences in brain function develop with cumulative neurotoxic exposure or precede stimulant initiation. Second, we examined whether comorbid THC use affected our findings by means of split group analyses as OSU frequently use cannabis (∆9 tetrahydrocannabinol, THC). College students reporting the abuse of prescription stimulants, for example, are 10 times as likely to report THC use than students not using stimulants [4]). Third, we quantified risk adjustment-related brain activation as a measure of learning from outcomes.

Four specific hypotheses were tested. First, consistent with studies of stimulant dependence, we hypothesized that OSU would exhibit lower DLPFC and SFG activation than CS while navigating risky and safe decisions. Second, based on literature examining decision making in stimulant addiction, we predicted that OSU would demonstrate lower ACC, IFG, insula, and striatal activation than CS during risky choices. Third, for the analysis focusing on the response to punishment, we predicted that those OSU who adjust performance after monetary loss towards safe responses would show stronger activation in areas associated with response selection and action monitoring (e.g. dACC [19, 38, 40]). Fourth, we anticipated that OSU would make a larger number of risky behavioral selections than CS [23]. Moreover, we expected that higher co-use of THC would be associated with more pronounced neurobehavioral impairments as previous work revealed dose-response effects for higher cognitive functions that require the integration of multiple cognitive abilities [18]. The prefrontal cortex poses a main target in that regard.

## Methods

### Subjects

The University of California San Diego (UCSD) Human Subjects Review Board approved the study protocol. OSU and stimulant naïve CS were recruited via flyers mailed to >7000 students at local universities, internet ads (e.g. Craigslist), and local university newspapers. Individuals were informed that this study aimed to examine behavior and brain functioning of people who use stimulants. To rule out an active high on stimulants, subjects were instructed to refrain from illegal substance use ≥72 hours before testing. Subjects gave written informed consent.

OSU were defined as having (1) at least n = 2 off-prescription uses of cocaine or prescription stimulants (prescription amphetamines, methylphenidate) over the past six months; (2) no evidence for lifetime stimulant dependence; (3) no lifetime use of methylphenidate or prescription amphetamines for medical reasons; (4) absence of treatment of substance or alcohol-related problems. The following exclusion criteria were applied for all subjects: (1) current (and past 6 months) diagnosis of major depressive disorder and anxiety disorders; (2) lifetime bipolar disorder and schizophrenia; (3) antisocial personality disorder and conduct disorder; (4) attention-deficit hyperactivity disorder (ADHD), (5) current positive urine toxicology test (exception due to long detectability: THC), (6) lifetime use of ecstasy >25 and (7) head injuries or loss of consciousness for >5min. Minimal methamphetamine use was not an exclusion criterion in OSU. Fifteen OSU endorsed a mean of n = 4.95 (mean, SD = 44.54) lifetime methamphetamine uses, with 11 of these OSU reporting fewer than n = 10 lifetime uses. Additional inclusion criteria for CS were no lifetime use of stimulants and no lifetime history of substance or alcohol related problems. Given frequently reported co-use of THC in stimulant users [4], lifetime THC use did not serve as an exclusion criterion in OSU. To allow for comparability with OSU, THC use was not an exclusion criterion for CS.

Out of 1214 individuals who participated in phone screens, 231 OSU and 63 CS completed an in-person clinical interview session. Experienced interviewers administered the Semi Structured Assessment for the Genetics of Alcoholism [48] and a detailed assessment of lifetime substance use using Timeline Follow-Back Methods [49]. 158 OSU (61 females, 97 males) and 50 CS (28 females, 22 males) met inclusion criteria and comprised the final sample. Participants’ diagnoses were confirmed in consensus meetings with a board-certified psychiatrist (M.P.P.) and trained study personnel. Groups did not differ on age or education (t-tests, p’s > 0.61, see Table 1) while relatively more female CS participated in the study. OSU reported to have initiated stimulant use at the age of 18.39±1.69 (m±SD) years. A total of 64 OSU and 3 CS reported minimal regular nicotine use. On average, OSU smoked a total of 6.55±4.89 cigarettes per day. All but three OSU and nineteen CS admitted lifetime THC use. OSU reported more alcohol use than CS. Scan day urine toxicology was available for 145 OSU and 48 CS and was THC-positive for 58 OSU and 1 CS. In OSU, number of lifetime stimulant uses correlated with lifetime THC use (r = 0.32, p<0.001) and THC use onset preceded initiation of stimulant use by 2.47±1.98 years (r = 0.39, p<0.001). In addition, 45 OSU met criteria for THC dependence (47% of these current dependence), whereas 47 OSU and 3 CS met criteria for lifetime THC abuse. OSU reported higher sensation seeking and impulsivity traits than CS. See Table 1 for sample characteristics and S1 Table for detailed self-reports on impulsivity and sensation seeking.

**Table 1:** Sample characteristics by group status.

	OSU n = 158	CS, n = 50	*t-test results* *	Low THC OSU, n = 32	High THC OSU, n = 42	CS,n = 46	One-way ANOVA results*, Direction THC Subgroups
	m (SD)	m (SD)		m (SD)	m (SD)	m (SD)	
**Sociodemographics**							
Age (years)	20.77 (1.54)	20.94 (2.12)	t_206_ = 0.518, p = 0.61	20.75 (1.59)	20.98 (1.63)	20.76 (1.93)	F_2,117_ = 0.217, p = 0.81
Education (years)	14.51 (1.33)	14.56 (1.49)	t_206_ = 0.241, p = 0.81	14.38 (1.39)	14.60 (1.35)	14.52 (1.50)	F_2,117_ = 0.223, p = 0.80
Females (%)	39%	44%	***0*.*03*** (χ^2^)	56%	33%	58%	***0*.*038***
^a^Estimated Verbal IQ	109.07 (7.26)	110.00 (6.94)	t_197_ = 0.790, p = 0.43	108.31 (7.69)	109.33 (5.61)	110.16 (6.65)	F_2,110_ = 0.692, p = 0.50
**Substance Use**							
Lifetime Cocaine Uses (n)	20.46 (36.99)	N/A		9.03 (15.37)	39.07 (52.50)	N/A	F_2,117_ = 17.,347, p = ***0*.*001*** ^d^
Lifetime Prescription Stimulant Uses (n)	25.39 (65.40)	N/A		22.38 (28.27)	43.76 (118.00)	N/A	F_2,117_ = 4.135, p = 0.263^d^
Lifetime Cannabis Uses (n)	900.68 (1417.41)	21.84 (87.06)	t_205_ = 7.723, p***<0*.*001***	19.25 (15.04)	2466.81 (1908.16)	4.02 (9.47)	F_2,117_ = 64.556, p = ***<0*.*001***, High THC OSU > CS and Low THC OSU
Number of Drinks in Preceding Week (n)	14.58 (13.99)	2.16 (4.52)	t_193_ = 9.368, p ***<0*.*001***	11.03 (10.69)	17.54 (15.75)	1.57 (3.79)	F_2,111_ = 22.101, p = ***<0*.*001***, High and Low THC OSU > CS
Typical Number of Weekly Drinks (n)	19.64 (14.56)	4.80 (3.49)	t_190_ = 11.382, p***<0*.*001***	16.28 (13.16)	22.39 (14.57)	4.6 (3.59)	F_2,103_ = 23.376, p = ***<0*.*001***, High and Low THC OSU > CS
Number of Current Smokers (n)	65%	6%		28%	64%	2%	
Lifetime Ecstasy Uses (n)	2.93 (4.80)	0.02 (0.14)	t_205_ = 7.588, p<***0*.*001***	1.42 (3.72)	5.33 (6.19)	0.00 (0.00)	F_2,116_ = 19.097, p = ***<0*.*001***, High THC OSU > CS and Low THC OSU
**Traits and Symptoms**							
^b^ ^,^ ^c^Number of ADHD Symptoms	1.64 (3.20)	0.94 (1.63)	t_134_ = 0.896, p = 0.37	1.34 (2.63)	2.26 (4.25)	2.39 (3.95)	F_2,117_ = 0.816, p = 0.44
^c^Number of Conduct Symptoms	1.60 (1.66)	0.62 (0.93)	t_206_ = 3.980, p***<0*.*001***	1.16 (1.53)	2.02 (1.69)	0.54 (0.91)	F_2,117_ = 12.473, p***<0*.*001***, High THC OSU > CS and Low THC OSU
Impulsivity (BIS total)	65.41 (9.46)	60.82 (6.70)	t_206_ = 3.186, p = ***0*.*002***	64.38 (10.19)	66.24 (8.57)	60.33 (6.74)	F_2,117_ = 5.672, p = ***0*.*004***, High THC OSU > CS
Sensation Seeking (SSS total)	24.98 (4.62)	19.54 (6.11)	t_206_ = 5.789, p***<0*.*001***	23.39 (4.38)	25.31 (4.58)	18.67 (5.35)	F_2,117_ = 21.815, p***<0*.*001***, High and Low THC OSU > CS
Symptoms of Depression (BDI total)	2.43 (3.12)	1.70 (2.67)	t_194_ = 1.405, p = 0.16	2.33 (3.03)	2.80 (3.05)	1.54 (2.66)	F_2,107_ = 1.914, p = 0.152

Abbreviations: OSU, occasional stimulant users; CS, stimulant naïve comparison subjects; THC, marijuana; Prescription Stimulants = Adderall, Ritalin (used without prescription); BIS, Barrett impulsiveness scale; SSS, sensation seeking scale, BDI, Beck depression inventory; ADHD, attention deficit hyperactivity disorder.

^a^ Verbal IQ estimated via WTAR or NAART.

^b^ Maximum number of ADHD symptoms = 21 (n = 10 attention deficit symptoms, n = 11 hyperactivity symptoms).

^c^ ADHD (age 6–13) and conduct (<18 years) symptoms as assessed during in-person clinical interview (SSAGA).

^d^ refers to t-test between Low and High THC OSU.

* Significance level of p<0.05.

### Risky Gains Task (RGT)

**Fig 1:**
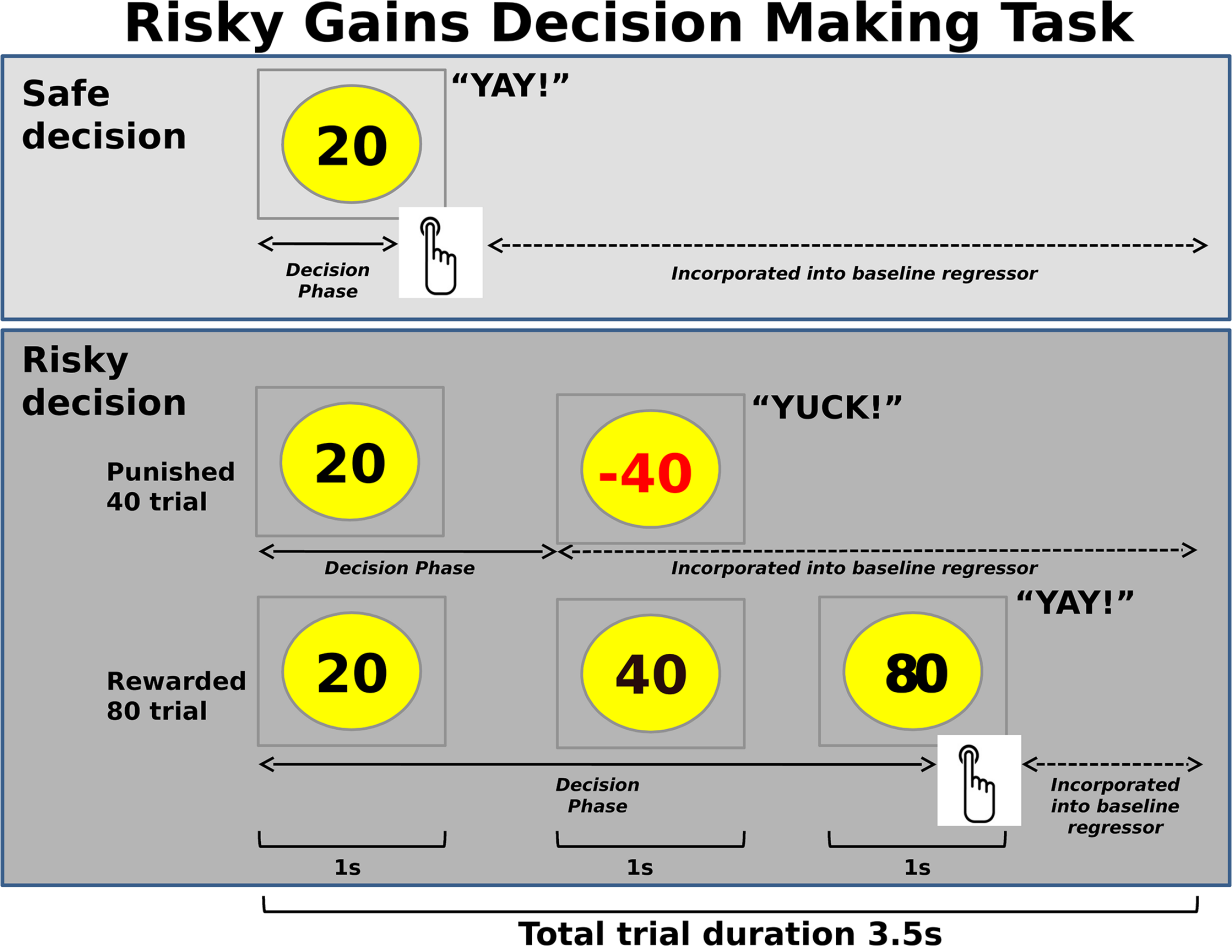
Risky Gains Task. In two subsequent gambles on 96 trials, subjects could gamble the safe option (cash in 20 cents) for double or nothing, to gain 40 or, in the potential second gamble, 80 cents (“risky” decisions). Positive values needed to be collected within their respective 1sec presentation window. 54 trials were predefined as rewarded (+20, +40, +80), 24 as punished 40 trials (-40) and 18 as punished 80 (-80) trials. Overall totals were displayed on top after a given trial, so that subjects could monitor their performance and monetary wins and losses. Decision phase regressors for fMRI analysis were defined as lasting from the onset of the trial until the subject had made a response, or, in the case of a response latency greater than 1sec, until the subject was presented with a negative value. The baseline regressor encompassed the time to initiation of the trial and the time after presenting the outcome, and also included the null trials that are interspersed.

The exact experimental task (Fig 1) has previously been used in our group [23, 50, 51]. Subjects were shown the numbers 20, 40 and 80 in ascending order (1s each), representing 20, 40 and 80 cents to be actually added to their total at the end of the experiment (gains of +20, +40, +80 cents). Participants were told that 20 was always the “safe option” but that they had the option to wait 1 second to receive 40 cents or wait for another second and receive 80 cents. However, they were told that there was a chance that 40 or 80 might come up in red, representing actual losses of money from the total to be paid at the end of the study (losses of -40, -80). 40 and 80 were explicitly referred to as “risky options”.

Unbeknownst to subjects, the frequency of a -40 or -80 outcome was predefined such that their final gain would be identical if they consistently selected 20, 40, or 80 cents. In other words, there was no inherent advantage of selecting risky over safe choices. Subjects were instructed that a positive value needed to be collected (index finger button press) in the respective 1s window. A press thereafter would result in a loss of the presented value. The length of 1s for this response window was chosen to even allow for potentially slow responding individuals to collect a desired option. Longer time frames may have encouraged relatively impatient or impulsive subjects to collect a given amount. Wins and losses were accompanied by auditory feedback (“yay” for wins and “yuck” for losses respectively). The overall total was displayed on top after the trial was completed so that subjects could monitor their performance and see the amount to be paid out in dollar at the end of the study.

The task comprised 96 trials, which lasted 3.5s regardless of subject’s response. Three trial types were presented in a pre-set randomized order: 54 rewarded trials (+20, +40, +80), and punished 40 (-40) and punished 80 (-80) trials. Besides potential losses due to subjects’ non- or slow responding on rewarded trials, the following setup resulted in different amounts of punished 40 and 80 trials per subject: 24 trials were predefined as punished 40 trials (-40), and 18 trials as punished 80 trials (-80). However, if a subject pressed to collect 20 on a trial meant to be a -40 trial, or tried to collect 20 or 40 on a dedicated -80 trial, the subject received the collected amount, therewith reducing the number of punished trials. 22 subjects (17 OSU, 5 CS) did not respond on one (n = 18 subjects) or more (n = 4 subjects, with a maximum of three missed responses, Table 2) of the rewarded trials, whereas the 188 remaining subjects responded in time on all 54 rewarded trials.

**Table 2:** Behavioral results on the risk taking task by group status.

	OSU (n = 158)	CS (n = 50)		Low THC OSU (n = 32)	High THC OSU (n = 42)	CS (n = 46)	
	m (SD)	m (SD)	t-test results*	m (SD)	m (SD)	m (SD)	*One-way ANOVA results* *
***Decision-making***							
**Safe Decisions**							
*Number of Safe Decisions (+20)*	43.55 (17.44)	43.50 (18.89)	t_206_ = 0.86, p = 0.93	48.25 (18.53)	44.62 (18.30)	43.85 (19.55)	F_2,117_ = 0.556, p = 0.58
**Risky Decisions**							
*% Risky Decisions*	50.22 (16.76)	50.00 (18.18)	t_206_ = 0.80, p = 0.94	45.78 (17.81)	49.19 (17.50)	49.94 (18.82)	F_2,117_ = 0.536, p = 0.59
*Number of Rewarded Risky Trials* ^**a**^ (n)	28.64 (9.98)	28.16 (10.62)	t_206_ = 0.291, p = 0.77	25.75 (10.19)	28.19 (10.62)	28.13 (11.04)	F_2,117_ = 0.598, p = 0.55
*Number of Punished Risky Trials* ^**b**^ (n)	17.93 (7.07)	18.00 (7.22)	t_206_ = 0.060, p = 0.95	16.44 (7.54)	17.79 (7.44)	18.02 (7.48)	F_2,117_ = 0.456, p = 0.63
***Response To Punishment***							
*% Risky Responses without prior Punishment*	53.04 (18.49)	52.15 (19.66)	t_206_ = 0.291, p = 0.77	47.68 (18.87)	52.20 (19.67)	52.09 (20.45)	F_2,117_ = 0.598, p = 0.55
*% Risky Responses after Punishment*	30.96 (19.30)	29.12 (18.14)	t_206_ = 0.597, p = 0.55	27.57 (22.80)	30.32 (17.96)	29.01 (17.67)	F_2,117_ = 0.185, p = 0.83

Abbreviations: OSU, occasional stimulant users; CS, stimulant naïve comparison subjects.

^a^ rewarded risky trials refers to +40 and +80 trials.

^b^ Punished risky trials refers to -40 and -80 trials.

* Significance level of p<0.05.

### Image Acquisition

The entire scanning session took about 60min and was preceded by a brief training session outside the MR scanner. The decision making task was implemented in a randomized fast-event related design, which was time-locked to the onset of 256 whole brain acquisitions (T2*-weighted EPI on a Signa EXCITE, GE Healthcare, Milwaukee, Wisconsin, 3T scanner; TR = 2000ms, TE = 32ms, FoV = 230x230 mm^2^, 64x64 matrix, 30 2.6-mm axial slices, 1.4mm gap, flip angle = 90°, duration: 8min, 32sec). Six resting trials were interspersed between 96 trials and ignored for analysis. During the same experimental session, a high-resolution T1-weighted image (TR = 8ms, TE = 3msec, FoV = 250x250 mm^2^, 192x256 matrix interpolated to a 256x256 matrix, flip angle = 12°, 172 sagitally acquired slices, .97x.97x1 mm^3^ voxels) was obtained for reference.

### Data Analysis

#### Grouping

Following comparisons of our main groups of interest (OSU, CS), effects of co-use of THC on decision making performance and related brain activation were addressed. Given that OSU and CS differed on lifetime THC use, it would be inappropriate to include it as a covariate [52]. Hence, OSU with high THC use (High THC-OSU, reporting >1000 lifetime uses and fulfilling THC abuse criteria, n = 42) were compared to OSU with low THC co-use (Low THC-OSU, reporting <50 lifetime uses, n = 32) and CS reporting <50 lifetime THC uses (n = 46). Comparison of OSU’s and CS’s sociodemographic data, substance use and traits and symptoms was compared by means of t-tests. Subgroup analyses were carried out in ANOVAs.

#### Behavioral analysis

The frequency of safe responses (+20) versus risky responses (+40; -40; +80; -80) as an index of risk-taking behavior was admitted to t-tests. We collapsed across all risky decisions to allow for a relatively even split between risky and safe decisions. T-tests were run to compare behavioral performance of OSU and CS, while ANOVAs were drawn on to compare 32 Low and 46 High THC-OSU with 46 CS reporting fewer than 50 lifetime THC uses. To analyze responses to monetary losses, the frequency of risky responses was examined as a function of preceding punishment (-40, -80).

#### Behavioral regressors for fMRI analysis

Five individual decision making regressors were constructed, starting from the onset of the trial (presentation of +20) and lasting until the subject made a response to collect 40 or 80 cents, until -40 or -80 were presented on dedicated punished trials or, in the case of a response latency greater than one second, until the subject was presented with a -40 or -80 (see Fig 1). Regressors reflected whether subjects (1) selected the safe response 20 (+20), (2) held out for 40 and gained 40 cents (+40), (3) held out for an 80 and gained 80 cents (+80), (4) held out for a 40 but lost 40 cents (-40), and (5) held out for 80 but lost 80 cents (-80).

#### fMRI data analysis

fMRI data were analyzed with AFNI (Analysis of Functional Neuroimages [53]).

*Preprocessing*. The temporal region with the largest span of fewest voxel-wise outliers was identified as a base for registration. Time series images were aligned to this base in dx, dy, dz, roll pitch and yaw directions. These adjustment parameters were used as nuisance regressors to account for movement. Data points with abnormally large amplitude relative to the surrounding time points were eliminated and interpolated. An automated coregistration of EPI and anatomical images was followed by censoring of additional time points exceeding the mean number of voxel outliers for the series. Additionally, experienced fMRI investigators (M.R., T.M.F.) inspected data sets for artifacts and sufficiency of alignment. Anatomical images were talairached by experienced investigators. Images were transformed into Talairach space.

*First level analysis*. Multiple regressor analysis and individual linear contrasts were set up in AFNI’s 3dDeconvolve. Specifically, deconvolution was performed examining the decision phase (risky decisions, safe decisions), including nuisance motion regressors (roll, pitch and yaw directions), a baseline (encompassing the time to initiation of the trial and the time after presenting the outcome; also including the interspersed null trials) and a linear drift regressor. Images were spatially smoothed applying a Gaussian filter 6mm full-width-half-maximum. The individual percent signal change (PSC) for risky (+40, -40, +80, -80) and safe (+20) decisions was calculated on a voxel-by-voxel basis throughout the entire brain and was obtained by dividing the coefficient of the regressor of interest by the baseline regressor.

*Second level analysis*. A linear mixed effects (LME [54]) analysis was conducted in R (http://cran.us.r-project.org/). Subjects were treated as random effects; group (OSU, CS) and decision (risky, safe) as fixed effects. The main effect group and the group by decision interaction served as the effects of interest to examine risk taking differences between OSU and CS. Whole brain analyses were performed and, following hypotheses, an additional region of interest (ROI) analysis was restricted to areas with particular relevance to risk taking, namely the caudate, insula and ACC. ROIs were defined based on the Talairach atlas [55, 56] and were combined into one “risk mask” comprising all ROIs. Analysis of fMRI data is usually preformed in a voxel-wise approach with all statistical tests conducted separately and simultaneously. Afni’s program AlphaSim [57], a threshold adjustment method based on Monte-Carlo simulations, was drawn on to guard against identification of false positive areas of activation. Specifically, AlphaSim takes the 6mm blur (see above) in conjunction with a cluster significance of *p*<0.05 into consideration. It identified a minimum volume of 1984μL for whole brain analyses to achieve a corrected p-value of *p*<0.05, while at least 640μL were required for the insula, 832μL for the ACC and 448μL for the caudate.

#### Secondary analyses

Three secondary analyses were conducted. First, the effect of cumulative substance use on brain activation was probed within OSU. Specifically, a Huber robust regression [58] was computed with lifetime use of stimulants and THC as predictors. The dependent measure was PSC during risky decisions. Lifetime stimulant use was natural log-transformed +1 due to non-normal distributions and was z-scored prior to regression entry. Second, the effect of lifetime THC co-use on brain activation was analyzed running an LME analogous to the main analysis: Low and High THC-OSU and CS were compared, focusing on the effects of group, decision (risky, safe) and the interaction group by decision. Third, a robust regression probing on risk adjustment-associated brain activation was run performed separately within CS and OSU. Here, the behavioral responses to punishment (percentage of risky and safe decisions following monetary loss) were predictors, wherein the dependent variable was PSC during risky and safe decisions. R2 was calculated on the peak cluster activation and was corrected via AlphaSim via t-values associated with each beta coefficient. A corrected p-value of .05 was applied.

## Results

### Behavioral Results

OSU and CS did not differ on their percentage of safe and risky responses or on their response to punishment, as reflected by a relative percentage of safe (+20) and risky (+40, -40, +80, -80) responses immediately following losses. Moreover, Low and High THC OSU and CS did not differ behaviorally (Table 2).

### Neuroimaging Results

#### Group main effect

**Fig 2:**
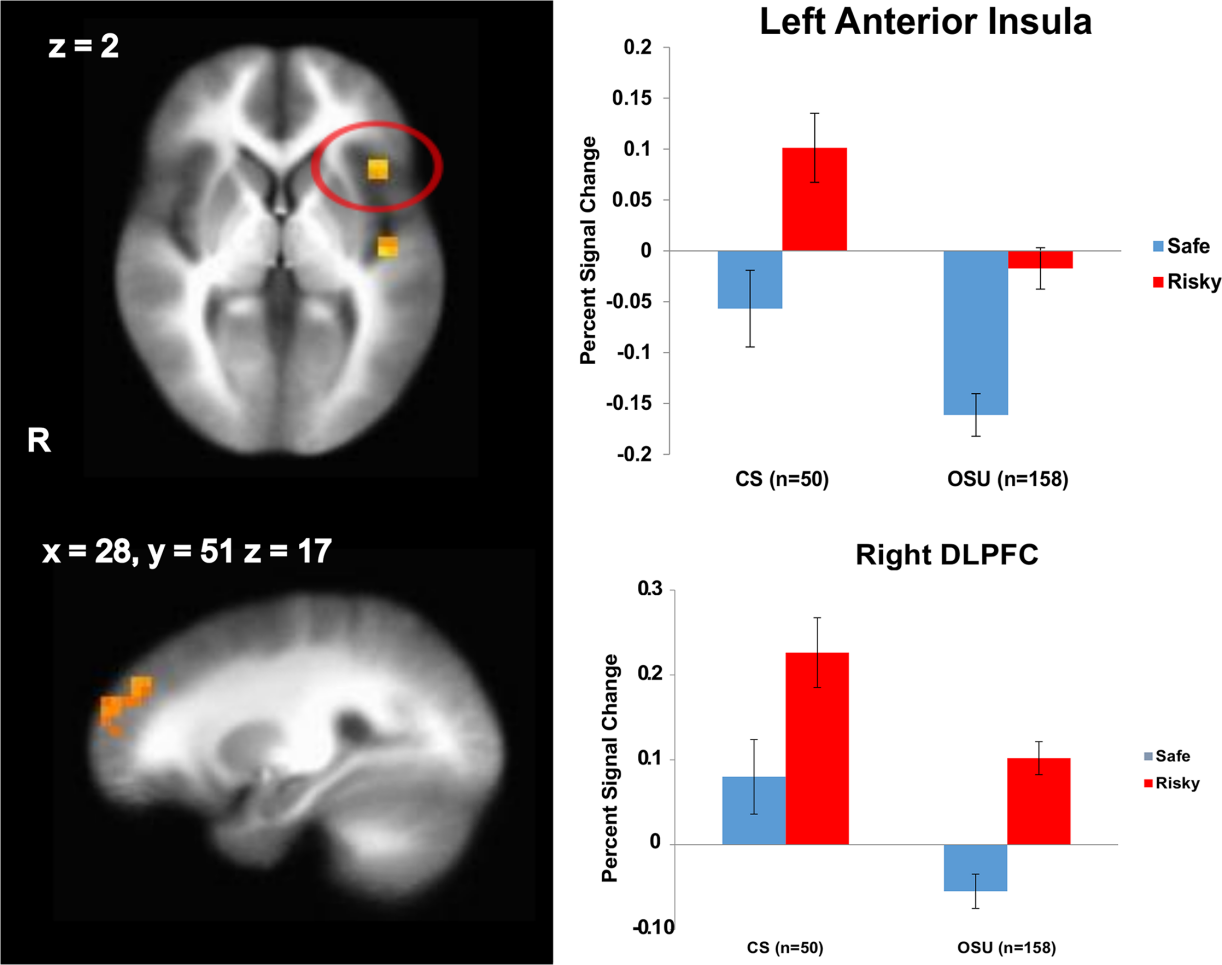
Occasional stimulant users (OSU) show an attenuated left anterior insula and right dorsolateral prefrontal (DLPFC) activation during risky and safe decision making. LME analysis results, group main effect (n = 208). Cluster significance of p < .05 corrected for multiple comparisons (voxel-wise probability: p < .05). R indicates right; error bars represent standard errors; CS = comparison subjects.

Results (Table 3) indicated that OSU showed attenuated activation in SFG, middle frontal gyrus (including DLPFC), dorsal striatum, and anterior/posterior insula compared to CS during decision making across risky and safe decisions (Fig 2). These findings were neither explained by use of alcohol, nicotine or THC, nor by self-reported impulsivity or sensation seeking tendencies as revealed by correlation analyses (S2 Table).

**Table 3:** fMRI linear mixed effects results for the group (OSU, CS) main effect (n = 208).

Region	L/R	Volume (μl)	x	y	z
^1^Superior / Middle Frontal Gyrus (including DLPFC; BA 10/46)	R	3264	28	51	17
^1^Middle Occipital Gyrus	L	2432	-40	-84	5
^1^Caudate	L/R	2048	-1	13	9
^2^Caudate	L	640	-5	12	7
^2^Posterior Insula	L	1024	-41	-13	2
^2^Anterior Insula	L	640	-39	18	2

For all regions of interest identified, CS showed stronger activations than OSU. Cluster significance of p < .05 corrected for multiple comparisons (voxel-wise probability: p < .05, F_1,156_ = 3.89).

Abbreviations: R, right; L, left; x, y and z: Brodmann Area (BA) coordinates; OSU, occasional stimulant users; CS, stimulant naïve comparison subjects; BA, Brodmann Area; SFG, superior frontal gyrus; DLPFC, dorsolateral prefrontal cortex.

^1^ Whole brain analysis.

^2^ Risk mask analysis.

#### Group by decision interaction

**Fig 3:**
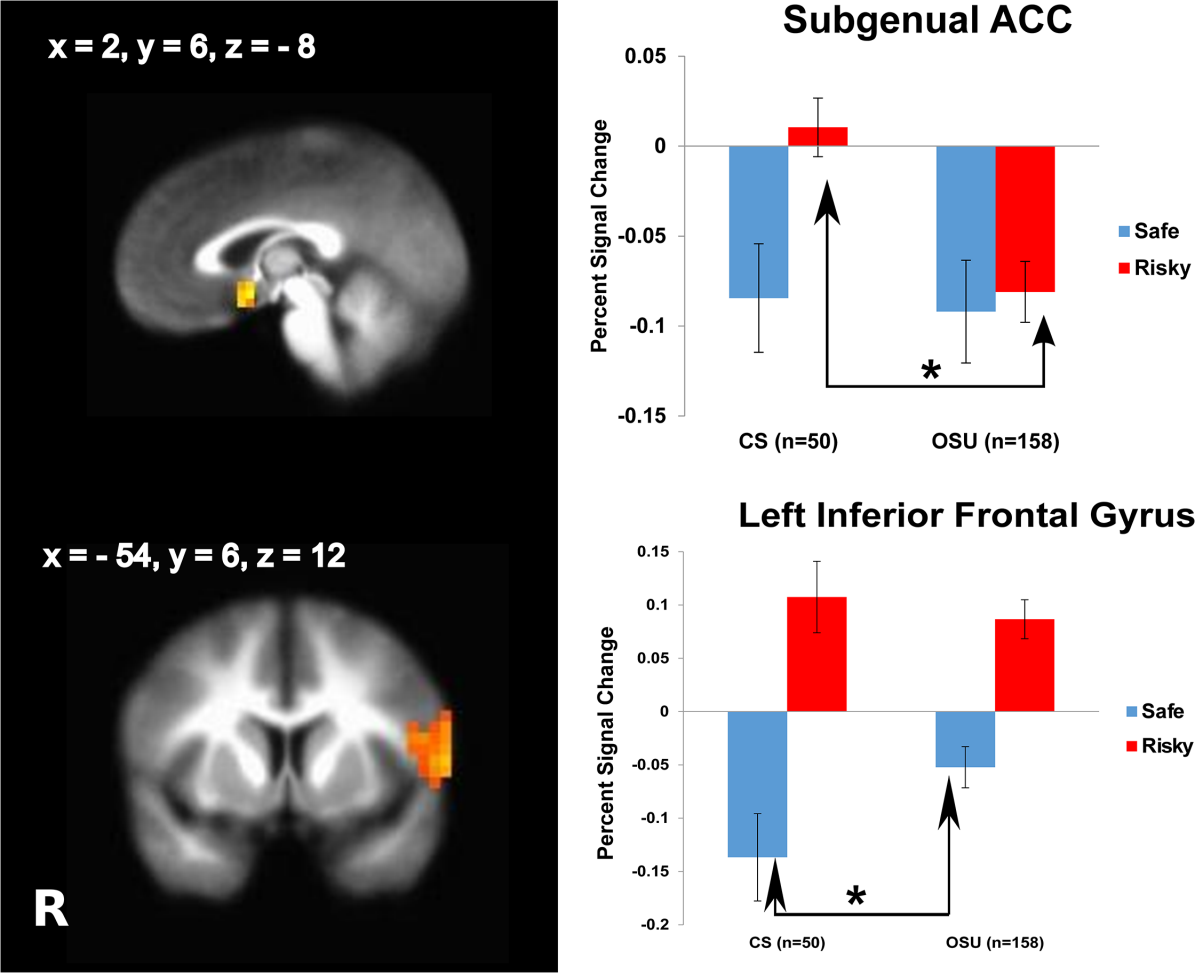
Occasional stimulant users (OSU) present lower subgenual anterior cingulate cortex (sgACC) recruitment during risky decisions and less deactivation of inferior frontal gyrus to safe decisions than comparison subjects (CS). LME analysis results, group by decision (risky vs. safe) interaction (n = 208). Cluster significance of p < .05 corrected for multiple comparisons (voxel-wise probability: p < .05). R indicates right, error bars represent standard errors, asterisk = sign. at p = 0.05.

Relative to CS, OSU displayed less activation during risky decisions in dorsal striatum, amygdala, hippocampus, sgACC and medial frontal gyrus (Table 4, Fig 3). In comparison, OSU showed greater activation during safe decisions in IFG, posterior cingulate, superior temporal gyrus and cuneus.

**Table 4:** fMRI linear mixed effects results for the group (OSU, CS) by decision (win risky vs. win safe) interaction (n = 208) .

Region	L/R	Volume (μl)	x	y	z	Win Risky Decisions	Win Safe Decisions
^1^Medial Frontal Gyrus (BA 6), Precentral/Postcentral Gyri	L/R	17,856	-1	-34	56	CS > OSU	ns
^1^Inferior Frontal Gyrus (BA 44/45), Precentral Gyrus	L	2880	-54	6	12	ns	OSU > CS
^1^Middle Temporal Gyrus, Lentiform Nucleus, Putamen, Amygdala, Hippocampus	L	13,184	-48	-35	-6	CS > OSU	ns
^1^Parahippocampal Gyrus, Thalamus	R	7872	24	-49	-6	ns	OSU > CS
^1^Lingual Gyrus	L	6656	-16	-45	-4	ns	OSU > CS
^1^Lingual Gyrus	L	5952	-5	-85	-8	CS > OSU	ns
^1^Inferior Temporal Gyrus	R	6464	51	-12	-20	CS > OSU	ns
^1^Superior Temporal Gyrus	R	3136	53	-57	16	ns	OSU > CS
^1^Cuneus	L	2432	-4	-71	21	ns	OSU > CS
^1^Posterior Cingulate	L	2304	-19	-62	13	ns	OSU > CS
^2^Subgenual ACC (BA 25)	R	1024	2	6	-8	CS > OSU	ns

Cluster significance of p < .05 corrected for multiple comparisons (voxel-wise probability: p < .05, F_1,156_ = 3.89).

Abbreviations: R, right; L, left; x, y and z: Brodmann Area (BA) coordinates; OSU, occasional stimulant users; CS; stimulant naïve comparison subjects; BA, Brodmann Area; IFG, inferior frontal gyrus; ACC, anterior cingulate cortex. Within each group, percent signal change for all Win Risky decisions was greater than Win Safe decisions.

^1^ Whole brain analysis.

^2^ Risk mask analysis.

#### Effect of lifetime stimulant use

**Fig 4:**
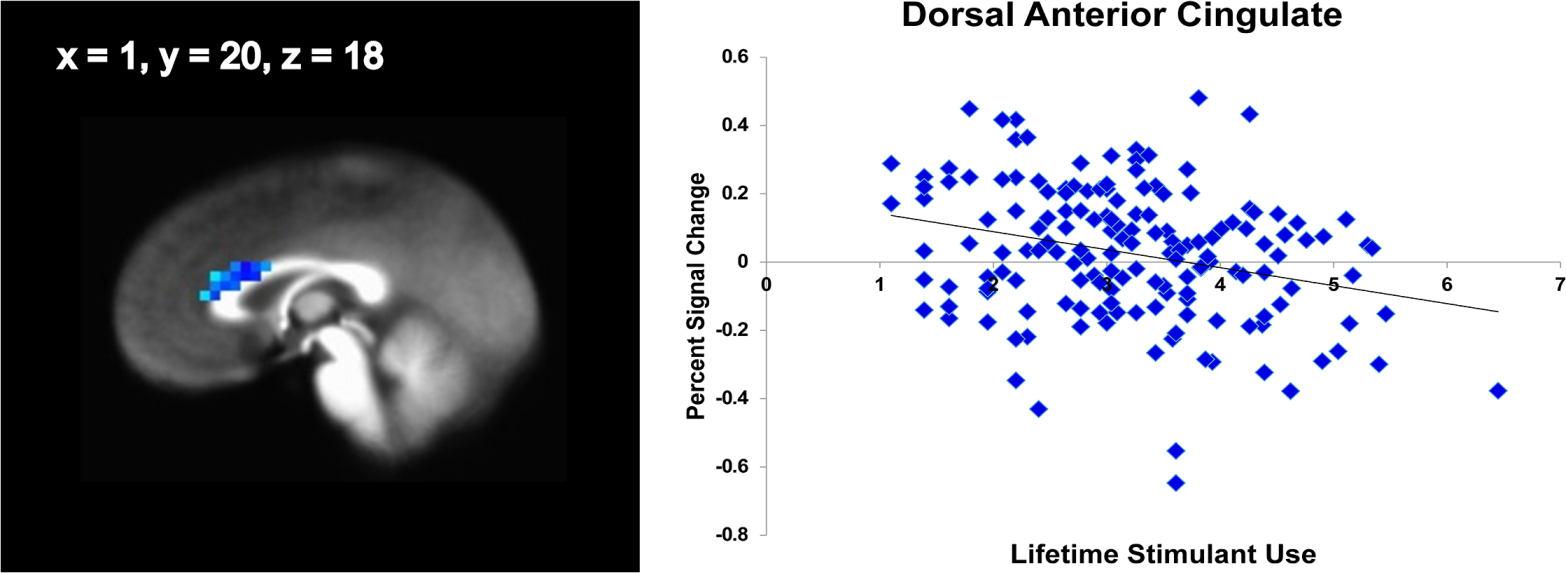
Huber robust regression with lifetime substance use in occasional stimulant users (n = 158). Stimulant users with relatively greater lifetime stimulant uses show a diminished recruitment of anterior cingulate. Risky vs. safe decisions, cluster significance of p < .05 corrected for multiple comparisons (voxel-wise probability: p < .05).

After controlling for THC use, OSU who had consumed relatively more stimulants showed relatively lower dACC (x = 1, y = 16, z = 23, 6080μl; x = 1, y = 20, z = 18, 3072μl, Fig 4) and posterior insula (x = -33, y = -17, z = 15, 768μl) but more left anterior insula (x = -44, y = -1, z = 1, 832μl) activation during risky decisions.

#### Effect of lifetime THC use

**Fig 5:**
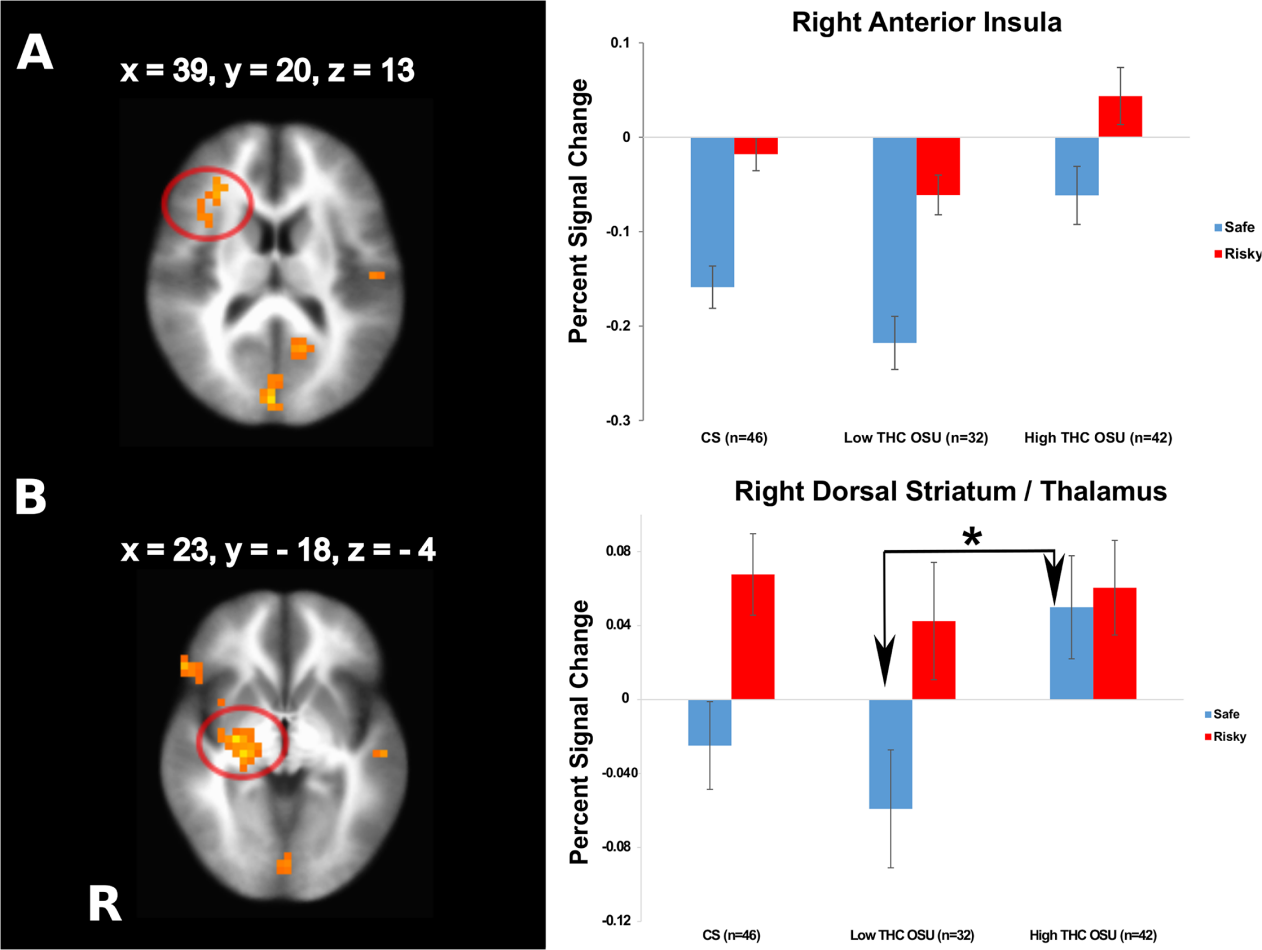
Effects of co-use of marijuana. Occasional stimulant users with relatively high numbers of lifetime co-use of marijuana (High THC OSU) are characterized by (A) a weaker relative deactivation of the right anterior insula to risky and safe decisions (main effect of subgroup,) and (B) a weaker neural differentiation of risky and safe decisions in the dorsal striatum compared to Low THC OSU driven by an absent relative decrease of activation during safe decisions (interaction subgroup by decision), n = 120. Cluster significance of p < .05 corrected for multiple comparisons (voxel-wise probability: p < .05). R indicates right, error bars represent standard errors, asterisk = sign. at p = 0.05.

Subgroup LME analyses identified brain regions affected by the amount of THC co-use. High THC-OSU exhibited greater striatum, DLPFC and anterior insula activation than Low THC-OSU and CS across risky and safe decisions as well as more parietal recruitment than Low THC-OSU and more posterior cingulate involvement than CS (Table 5, Fig 5A). Moreover, High THC-OSU presented relatively more striatal, IFG, medial and middle frontal and superior temporal activation than Low THC-OSU during safe decisions only (interaction subgroup by decision, Table 6, Fig 5B) leading to a deamplified neural differentiation of risky and safe decisions.

**Table 5:** fMRI linear mixed effects results for the group (low THC-OSU, high THC-OSU, low THC-CS) main effect (n = 120).

Region	L/R	Volume (μl)	x	y	z
**High THC OSU showing stronger activation than CS and Low THC OSU**					
Lentiform Nucleus, Putamen	R	3776	24	10	-3
Middle Frontal Gyrus (incl. DLPFC), Precentral Gyrus	R	2496	44	24	36
Anterior Insula	R	2048	39	20	13
**High THC OSU showing stronger activation than Low THC OSU**					
Cuneus	R	3328	2	-82	14
Inferior Parietal Lobule	L	2048	-50	-27	24
**High THC OSU showing stronger activation than CS**					
Posterior Cingulate	L	1984	-15	-53	11

Whole brain analysis results. Cluster significance of p < .05 corrected for multiple comparisons (voxel-wise probability: p < .05, F_2,117_ = 3.07).

Abbreviations: R, right; L, left; x, y and z: Brodmann Area (BA) coordinates.

**Table 6:** fMRI linear mixed effects results for the group (low THC-OSU, high THC-OSU, low THC-CS) by decision (win risky vs. win safe) interaction (n = 120).

Region	L/R	Volume (μl)	x	y	z	Win Safe Decisions
^1^Parahippocampal Gyrus, Superior Temporal Gyrus	L	4224	-25	-22	-17	High THC OSU > Low THC OSU
^1^Superior Temporal Gyrus	R	4224	47	-58	28	
^1^Middle Temporal Gyrus	L	3904	-47	5	-25	
^1^Superior, Middle, and Inferior Temporal Gyrus	R	2112	44	1	-30	
^1^Lentiform Nucleus, Globus Pallidus, Thalamus	R	3648	24	-18	-4	High THC OSU > Low THC OSU
^1^Declive	R	4224	1	-78	-12	
^1^Postcentral Gyrus	L	3968	-27	-32	58	
^1^Paracentral Lobule	L	2176	-1	-25	51	
^1^Subcallosal Gyrus	R	2176	27	4	-10	High THC OSU > Low THC OSU
^1^Inferior Frontal Gyrus	R	2176	43	24	-7	High THC OSU > Low THC OSU
^1^Medial Frontal Gyrus	L/R	1984	-2	45	27	High THC OSU > Low THC OSU
^1^Middle Frontal Gyrus	R	1984	18	4	59	High THC OSU > Low THC OSU

Cluster significance of p < .05 corrected for multiple comparisons (voxel-wise probability: p < .05, F_2,117_ = 3.07).

Abbreviations: R, right; L, left; x, y and z: Brodmann Area (BA) coordinates; ACC, anterior cingulate cortex; SFG, superior frontal gyrus; negative, negative correlation between lifetime use and BOLD; positive, positive correlation between lifetime use and BOLD.

^1^ Whole brain analysis.

#### Brain Behavioral Relationships

Table 7 summarizes brain regions associated with risk-taking as a function of preceding losses as identified in whole brain and risk mask analyses respectively. In CS, individuals who were more likely to respond with selecting a safe option after monetary loss also had greater activation in dorsal striatum (R^2^ = 0.14), dACC (R^2^ = 0.054) and cuneus (R^2^ = 0.105) when they selected a safe option. A similar relationship was observed for OSU in the dorsal striatum (left, R^2^ = 0.011; right, R^2^ = 0.016). Moreover, OSU who selected a safe response relatively more often after losses also had greater activation in dACC (R^2^ = 0.017) and IFG (R^2^ = 0.008) when choosing this option. In contrast, OSU who selected risky options relatively more often after punishment displayed less activation in an area extending from the dorsal striatum to sgACC (R^2^ = 0.048), as well as IFG (BA 44, R^2^ = 0.059) and posterior insula (R^2^ = 0.038).

**Table 7:** Huber robust regressions with behavioral responses (% of risky and safe decisions) after punishment predicting brain activation during risky and safe decision making. In CS, no clusters survived the threshold for prediction PSC during risk taking based on % risky decisions after punishment.

Group	Region	L/R	Volume (μl)	x	y	z	Direction
***% Risky Responses After Punishment Predicting PSC for Risky Decisions***							
OSU*	^1^Lentiform Nucleus, Caudate, Subgenual ACC (BA 24)	L	2752	-9	3	-8	negative
	^1^IFG (BA 44)	R	2176	-48	6	20	negative
	^1^Fusiform Gyrus	L	21,312	41	-49	-5	negative
	^1^Precentral Gyrus	L	10,368	-47	-21	34	negative
	^1^Middle Temporal Gyrus	L	7872	-49	-62	3	negative
	^1^Declive	L	5184	-46	-52	-19	negative
	^1^Cuneus	L	5184	-9	-80	18	negative
	^1^Culmen	R	4608	0	-60	4	negative
	^1^Thalamus	R	3648	5	-12	6	negative
	^1^Dorsal ACC (BA 24)	L	2816	4	-8	32	negative
	^1^Precuneus	R	2204	-2	-59	62	negative
	^2^Posterior Insula	L	704	41	-17	3	negative
	^2^Caudate	R	576	-9	14	-4	negative
	^2^Caudate	L/R	512	6	7	6	negative
***% Safe Responses After Punishment Predicting PSC for Safe Decisions***							
CS**	^1^Caudate/Middle Temporal Gyrus/ Hippocampus/Parahippocampus	R	4224	32	-43	6	positive
	^1^Dorsal ACC/Middle Frontal Gyrus	R	3648	21	1	38	positive
	^1^Cuneus	R	3200	4	-78	21	positive
OSU*	^1^Precentral/Postcentral Gyrus	L	11,136	-35	-25	49	positive
	^1^Cuneus	L	2432	-5	-80	12	positive
	^1^Parahippocampal Gyrus	L	2112	-7	-38	3	positive
	^1^IFG/Middle Frontal Gyrus (BA 9)	R	2112	49	18	26	positive
	^1^Dorsal ACC (BA 24)	L/R	1984	2	2	31	positive
	^2^Caudate	L	768	-6	14	-2	positive
	^2^Caudate	R	640	8	5	8	positive

Cluster significance of p < .05 corrected for multiple comparisons (voxel-wise probability: p < .05, critical t-value: 1.98, *df = 1, 157, **df = 1, 49).

Abbreviations: R, right; L, left; x, y and z: Brodmann Area (BA) coordinates; OSU, occasional stimulant users; CS; stimulant naïve comparison subjects; ACC, anterior cingulate cortex; IFG, inferior frontal gyrus; SFG, superior frontal gyrus; DLPFC, dorsolateral prefrontal cortex; negative, negative correlation between response to punishment and BOLD; positive, positive correlation between response to punishment and BOLD; PSC, percent signal change.

^1^ Whole brain analysis.

^2^ Risk mask analysis.

## Discussion

This study examined whether individuals who put themselves at high risk for stimulant dependence by using stimulants occasionally, show behavioral and/or neural processing differences in risk-taking decision making situations and yielded three main findings. First, OSU did not differ from healthy comparison subjects on their preference for risky versus safe options. Second, OSU showed attenuation of brain activation in areas that are important for risk processing as well as reduced neural differentiation of risky and safe options in brain areas essential for cognitive control mechanisms and bodily representation of risk. Third, THC use did not modulate these findings, but excessive co-use had additional effects on neural activation, which could contribute to poorer processing of risk.

In line with our hypothesis based on stimulant dependent individuals [39, 45, 59, 60], OSU exhibited insula and DLPFC attenuation across safe and risky decision making. Moreover, OSU relative to CS showed lower sgACC and striatal activation during risky decisions, but smaller IFG deactivation during safe decisions. OSU with greater lifetime stimulant use exhibited lower dACC and posterior insula activation during risky decision making. Finally, higher amounts of THC co-use were associated with a less pronounced deactivation and less neural differentiation of risky and safe options within the striatum, insula and prefrontal cortex. OSU who adjusted behavior towards lowered risk as a response to monetary losses resembled CS [26, 28, 29, 61] by exhibiting dorsal striatum, IFG, and dACC activation during decision making more than OSU who engaged in continued risk. Taken together, these results support the hypothesis that individuals even at very early stages in their stimulant use show aberrant risk-related brain activation towards recruiting brain areas less that are important for cognitive control or interoceptive processing. Attenuated neural differentiation of risky and safe options may lead an individual to process risky options and/or associated outcomes differently. This blunted neural response especially in brain areas essential for cognitive control mechanisms may make it difficult for OSU to refrain from risky decisions.

Given that the present study design was cross-sectional and did not include subjects prior to any stimulant use in a follow-up design, we cannot strictly conclude that these differences precede the onset of stimulant use. Nevertheless, because OSU had minimal exposure to stimulants and the majority of brain differences were not affected by THC use, it is most likely that these differences are not a consequence of extended stimulant use. Taken together, we identified brain activation patterns in OSU typical for chronic users [38–40, 45, 60, 62, 63] at stages where subjects–in this case high functioning students in challenging academic situations–and their environment, e.g. school, work and health care system, may not be aware of this heightened risk as overt behavioral impairments are not yet prominent. Given the minimal exposure to neurotoxic substances in our sample, we hypothesize that these may have preceded stimulant initiation. Longitudinal studies starting in stimulant naïve individuals are needed to verify this hypothesis.

Behaviorally, OSU performed comparably to CS, which is at odds with our prior investigation using the risky gains task in a small sample of OSU [23]. One possible explanation for this contradiction is the fact that only students without evidence for ADHD or antisocial personality disorder were included into the current investigation. The absence of behavioral differences is in line with our previous findings of absent or only subtle cognitive impairments on standard neuropsychological tests [64, 65] and other neuroimaging tasks [66, 67] in this sample. The absence of behavioral alterations at early stages of stimulant use suggests that cognitive impairments may increase with greater use of drugs [22, 68] as research has highlighted behavioral impairments in chronic users. Nevertheless, neural differences emerged between OSU and CS despite the absence of behavioral impairments in OSU, suggesting that OSU and CS differ in the process arriving at decisions. Specifically, OSU presented an attenuated neural differentiation of risky and safe decisions, thus seemed less sensitive to adverse consequences of risk taking. This finding extends recent results of our group showing a blunted neural response to risk in the absence of performance differences to be indicative of relapse to stimulant use [50].

OSU displayed a pronounced attenuation of dorsal striatum, ACC, prefrontal and insular activation to both risky and safe decisions. Drawing on findings in healthy subjects and stimulant dependence [26, 27, 36, 37, 44, 69], these results suggest that neural decision making impairments in OSU relate to dysfunctions in all aspects of decision making. First, OSU showed attenuated recruitment of prefrontal cortex, ACC, ventral striatum and left anterior insula. These areas are, among others, crucial for various cognitive rather than motivational/emotional aspects of decision making, e.g. discerning reward values of available choices, maintaining task objectives in short term memory, calculation of risk prediction error or choice-outcome contingency learning. Thus, these findings are similar to findings observed in stimulant dependent subjects. Specifically, chronic cocaine and methamphetamine dependent patients in contrast to non-users have for instance been shown to exhibit DLPFC and SFG hypoactivation for instance during delay discounting tasks, which require subjects to weigh alternatives with immediate or short-term outcomes against decisions with higher, but delayed pay offs while making decisions during hard compared to easy trials [39, 62] as well as DLPFC reductions during decision making [45, 60]. Prior work also demonstrates IFG reductions in stimulant dependent individuals during decision making [40–43]. Our findings now suggest that decision making related attenuation of prefrontal recruitment is prominent at very early stages of stimulant use, suggestive of a neural pattern potentially promoting initiation of stimulant use.

Second, OSU were also characterized by attenuated activation of the dACC, a brain area crucial for detection of conflict and action selection. Moreover, OSU presented attenuated rACC and sgACC activation, which have been related to motivational and learning-related aspects of goal-directed behavior [32–34]. Our findings in OSU extend prior work in chronic users, where cocaine dependent patients showed reduced ACC activation across risky and safe conditions while gambling [38]. Multi-substance abusers also exhibited attenuated ACC activation, which correlated with a greater number of risky decisions [19]. Third, attenuation of striatal, sgACC and insula activation support the notion that OSU experience risk as less aversive than CS as revealed by research on non-users with different risk preferences [70]. The attenuation also suggests a diminished linkage of risk with positive and negative outcomes in OSU. Risk-outcome associations, however, are essential for successful behavioral adjustment in terms of subsequent avoidance of punishment. Prior work in chronic substance users revealed diminished caudate activation to a variety of non-drug rewards [46, 47], findings summarized as the ‘reward deficiency syndrome’ hypothesis. Our findings of attenuated insula activation in a substantial sample of OSU are in line with previous decision making studies in cocaine dependent patients [40], relapsing methamphetamine dependent individuals [63], and OSU [71], whereas multi-substance abusers showed greater insula activation to higher number of risky choices [19]. Together, these patterns of neural attenuation in OSU in brain regions required for successful goal-directed decision making and generation of an interoceptive awareness to generate internal alarm signals to avoid risk may in the end promote continued risk-taking like drug use.

Secondary analyses verified that THC, which was substantially consumed by some of our participants, did not directly influence these activation patterns. While THC is known to promote cognitive and neural impairments [72–74], co-use of THC was not an exclusion criterion, as research shows that more than half of stimulant using students consume THC [4]. The onset of THC use in our sample typically preceded stimulant initiation. THC had additive neural effects, in that OSU with heavy THC use were characterized by a lacking differentiation of risky and safe options in related brain areas critical for decision making (e.g. right anterior insula and DLPFC), supporting studies revealing dose-dependent THC effects on decision making and brain activation [18].

Importantly, higher lifetime stimulant use was associated with diminished risk-taking activation in dACC and posterior insula after controlling for THC co-use. Our dACC findings demonstrate that OSU with relatively more uses show more pronounced neural decision making alterations in areas associated with cognitive control functions like response selection, proactive performance monitoring and conflict monitoring [30], findings consistent with research in chronic users [75–77]. The posterior insula has been shown to play a crucial role in the generation of interoceptive awareness [36]. The present finding of reduced activation in those OSU with higher lifetime uses may thus reflect diminished bodily representations of risk. The perception of (external and) internal alarm signals is crucial for the avoidance of risky behavior. While we cannot ultimately differentiate whether OSU with initially more pronounced brain functional alterations consumed more stimulants in a self-medication approach or whether repeated exposure to neurotoxic substances resulted in changes in neural activation patterns, dose-dependency and self-medication hypotheses find support in previous research showing that more frequent cocaine use leads to more pronounced dACC hypoactivations [78] whereas acute administration of cocaine fosters normalization of ACC hypoactivity [79]. To summarize, our dose-related findings suggest that both an attenuation of cognitive control mechanisms and bodily awareness foster prolonged exposure to risk as neither cognitive nor internal alarm signals support inhibition of prepotent responses.

Our results focusing on performance following losses revealed that OSU who took lower risks after monetary losses were characterized by a successful recruitment of brain structures CS employ to monitor conflict (dACC) and link outcomes with choices (dorsal striatum). On the contrary, OSU who repeatedly engaged in risk by selecting risky options showed a diminished activation of the sgACC, which is crucial for motivational aspects of decision making. OSU also presented alterations of dorsal striatum activation. With the dorsal striatum being engaged in mechanisms avoiding choices associated with losses, the findings suggest an attenuated emotional representation of risk, which may make it difficult for stimulant users to avoid risky choices. They may expose themselves to continued risk (e.g. in terms of continued substance use), which in the end might lead to a higher likelihood to transition to dependence. Cognitive-behavioral, pharmacological and/or neurofunctional trainings aimed at enhancing activation in those target areas might enable individuals to re-gain control over risk taking.

In summary, findings demonstrate that individuals at very early stages of stimulant use, similar to stimulant dependent individuals, show attenuated neural processing of risk and particularly less pronounced neural differentiation of risky and safe options. Given minimal exposure to stimulants in our sample, these deficiencies may have preceded stimulant initiation and might serve as vulnerability markers to allow for early intervention. While not directly linked, neural deficiencies were potentially promoted by THC use as particularly excessive THC users showed attenuated neural differentiation of risky and safe options in related but distinct networks. Through this mechanism, early THC use may have lowered the threshold for stimulant initiation. Stimulant use was associated with aberrant activation in brain areas crucial for cognitive and motivational aspects of goal-directed decision making. In conjunction with diminished bodily representations of risk weakening internal warning signals, these characteristics might foster continued risk-taking and thus increase likelihood for prolonged consumption and transition to dependence. Critical next steps will be the concrete delineation of risk factors for the stimulant-using individual and longitudinal studies preferably starting with individuals prior to any substance use. Such studies might confirm the hypothesized scenario of neural activation patterns encouraging continued substance use.

This study has some limitations. First, our study design was cross-sectional and not completely prospective; we included stimulant using individuals with very limited exposure to stimulants lacking clinically significant stimulant-related problems. This design makes it difficult to differentiate between pre-existing characteristics and effects of repeated exposure to drugs. Nevertheless, the mean number of stimulant exposure was minimal, making neurotoxic effects of stimulants less likely while supporting the hypothesis of a neural vulnerability predisposing to stimulant initiation. Research in stimulant naïve subjects at high risk for stimulant use (e.g. young first degree relatives of stimulant dependent individuals) may separate a genetic vulnerability for stimulant related problems from factors associated with occasional use. Second, co-use of THC did not serve as an exclusion criterion to allow for a representative sample of stimulant using young individuals [4]. While the current design did not include a subgroup of non-stimulant using THC users, we approached a potential THC effect by split group analyses and revealed that THC did not directly influence effects. THC had additive neural effects attenuating neural differentiation of risky and safe responses. Longitudinal studies including individuals prior to any substance use and the comparison with pure THC users are warranted to tease apart effects of THC and stimulants. We specifically excluded stimulant using and comparison subjects with a lifetime history (diagnosis or treatment) of ADHD and conduct disorder, both of which known to contribute to the propensity of substance use [80]. These strict exclusion criteria may also explain why we could not replicate previous findings of Leland and Paulus [23] administering the very same task to a smaller sample of OSU. Taken together, we feel confident that our results of attenuated neural representation of risk-related decision making in OSU delineate precursors of stimulant use problems. Future analyses will focus on the concrete predictive value for the stimulant-using individual. Third, the task was set up in a way that there was no inherent advantage of selecting safe or risky options to lead to similar monetary wins across subjects. While participants were instructed that holding out for 40 or 80 were “risky options”, this implementation did not encourage contingency learning, as no underlying optimal strategy was reinforced. Lastly, it needs to be discussed whether predetermined temporal aspects of our task may have influenced results. In this regard, it is important to point out that potential differences in individual response times are unlikely to have affected our results. The time window during which subjects needed to collect a value displayed on the screen was set to 1s, so that potentially slow responding subjects were able to collect a given amount. On the other hand, this window of opportunity (see [23] for a more detailed discussion) added a certain time pressure to our task, so that waiting was disadvantageous. One might also argue that our behavioral options not only differed on risk domain but also on waiting domain, as subjects always had to wait longer to gain higher rewards, which increased the chances of higher losses. Hence, results could technically also be driven by differences in subjects’ willingness to wait, particularly given that OSU and CS differed on some self-reported measures of impulsiveness. Our results (see S1 Table) however verify, that group differences of brain activation were not explained by differences in self-reported impulsivity. In addition, our task was set up in a way that longer waiting would have resulted in monetary losses. Groups did not differ on the number of missed trials so that we are confident, that our results are not related to a differential willingness to wait.

## Supporting Information

S1 Table Self-reported impulsivity and sensation seeking. BIS and SSS subscale group and subgroup differences, n = 208. Abbreviations: BIS, Barrett impulsiveness scale; SSS, sensation seeking scale.
(DOCX)

S2 Table Attenuation of occasional stimulant users’ (OSU) activation of anterior and posterior insula and caudate as revealed by LME main effect group is neither explained by co-use of alcohol, nicotine or marijuana, nor by self-reported impulsiveness (BIS) or sensation seeking (SSS). Data given are p-values deriving from correlations. Following Bonferroni corrections for multiple comparisons, correlations were considered significant in case of p<0.004 (12 variables, 0.05/12 = 0.004). For BIS and SSS, only those subscales were subjected to correlation analyses that differed between OSU and CS (see Table 1).
(DOCX)
