# Supplementary material for: Attenuated Neural Processing of Risk in Young Adults at Risk for Stimulant Dependence
Source: PLoS One. 2015 Jun 15;10(6):e0127010. doi: 10.1371/journal.pone.0127010 (PMC4468216; doi:10.1371/journal.pone.0127010)
Supplement: S1 Table — BIS and SSS subscale group and subgroup differences, n = 208. Abbreviations: BIS, Barrett impulsiveness scale; SSS, sensation seeking scale. (DOCX) [file pone.0127010.s001.docx]

**Supplementary Table I. Self-reported impulsivity and sensation seeking.** BIS and SSS subscale group and subgroup differences, n=208

**R**

Abbreviations: BIS, Barrett impulsiveness scale; SSS, sensation seeking scale.

|  | **OSU  n=158** | **CS,  n=50** | ***t-test results**** | **Low THC OSU, n=32** | **High THC OSU, n=42** | **CS, n=46** | **One-Way ANOVA THC Subgroups results*** |
| --- | --- | --- | --- | --- | --- | --- | --- |
|  | **m (SD)** | **m (SD)** |  | **m (SD)** | **m (SD)** | **m (SD)** |  |
| **Impulsivity (BIS total)** | 65.41 (9.46) | 60.82 (6.70) | t_206_=3.186, p=***0.002*** | 64.38 (10.19) | 66.24 (8.57) | 60.33 (6.74) | F_2,117_=5.672, p=***0.004***, High THC OSU > CS |
| *BIS Subscale Attention* | 10.76 (2.57) | 9.79 (2.24) | t_206_=2,405, p=***0.02*** | 10.70 (2.68) | 10.85 (2.29) | 9.58 (2.10) | F_2,117_=3.846, p=***0.024***, High THC OSU > CS |
| *BIS Subscale Motor* | 16.33 (3.25) | 14.56 (2.60) | t_206_=3,498, p=***0.001*** | 16.12 (3.66) | 16.60 (3.13) | 14.57 (2.67) | F_2,117_=5.090, p=***0.008***, High THC OSU > CS |
| *BIS Subscale Self Control* | 13.03 (2.84) | 12.35 (2.31) | t_206_=1.529, p=0.13 | 12.22 (2.63) | 13.29 (2.67) | 12.17 (2.90) | F_2,117_=2.620, p=0.077 |
| *BIS Subscale Cognitive Complexity* | 11.54 (2.23) | 11.16 (1.98) | t_206_=1.10, p=0.27 | 11.92 (2.15) | 11.74 (2.38) | 11.13 (2.04) | F_2,117_=1.469, p=0.234 |
| *BIS Subscale Perseverance* | 7.59 (1.82) | 6.94 (1.63) | t_206_=2.272, p=***0.02*** | 7.03 (1.74) | 7.77 (1.82) | 6.87 (1.68) | F_2,117_=2.926, p=0.058 |
| *BIS Subscale Cognitive Instability* | 6.12 (1.66) | 6.04 (1.81) | t_206_=0.288, p=0.77 | 6.01 (1.78) | 5.97 (1.70) | 6.05 (1.86) | F_2,117_=0.018, p=0.982 |
| **Sensation Seeking (SSS total)** | 24.98 (4.62) | 19.54 (6.11) | t_206_=5.789, p***<0.001*** | 23.39 (4.38) | 25.31 (4.58) | 18.67 (5.35) | F_2,117_=21.815, p***<0.001***, High and Low THC OSU > CS |
| *SSS Subscale Thrill and Adventure Seeking* | 7.92 (1.92) | 7.46 (2.43) | t_206_=1.239, p=0.22 | 7.46 (2.35) | 7.90 (1.51) | 7.32 (2.48) | F_2,117_=0.820, p=0.443 |
| *SSS Subscale Experience Seeking* | 6.58 (1.71) | 5.28 (1.87) | t_206_=4.591, p***<0.001*** | 5.96 (1.68) | 6.76 (1.58) | 5.11 (1.78) | F_2,117_=10.533, p***<0.001,*** High THC OSU > CS |
| *SSS Subscale Disinhibition* | 6.78 (2.10) | 3.80 (2.66) | t_206_=8.168, p***<0.001*** | 6.54 (2.07) | 6.91 (2.40) | 3.48 (2.51) | F_2,117_=27.397, p***<0.001***, High and Low THC OSU > CS |
| *SSS Subscale Boredom Susceptibility* | 3.47 (1.98) | 2.84 (1.84) | t_206_=1.997, p=***0.047*** | 3.27 (2.15) | 3.48 (1.89) | 2.58 (1.58) | F_2,117_=2.794, p=0.065 |

* Significance level of p<0.05.
