# Supplementary material for: Attenuated Neural Processing of Risk in Young Adults at Risk for Stimulant Dependence
Source: PLoS One. 2015 Jun 15;10(6):e0127010. doi: 10.1371/journal.pone.0127010 (PMC4468216; doi:10.1371/journal.pone.0127010)
Supplement: S2 Table — Data given are p-values deriving from correlations. Following Bonferroni corrections for multiple comparisons, correlations were considered significant in case of p<0.004 (12 variables, 0.05/12 = 0.004). For BIS and SSS, only those subscales were subjected to correlation analyses that differed between OSU and CS (see Table 1). (DOCX) [file pone.0127010.s002.docx]

**Supplementary Table II. Attenuation of occasional stimulant users’ (OSU) activation of anterior and posterior insula and caudate as revealed by LME main effect group is neither explained by co-use of alcohol, nicotine or marijuana, nor by self-reported impulsiveness (BIS) or sensation seeking (SSS).** Data given are p-values deriving from correlations. Following Bonferroni corrections for multiple comparisons, correlations were considered significant in case of p<0.004 (12 variables, 0.05/12=0.004). For BIS and SSS, only those subscales were subjected to correlation analyses, that differed between OSU and CS (see Table 1).

|  | **SFG/Middle Frontal/DLPFC/BA 10/46^a^** | **Middle Occipital^a^** | **Caudate^a^** | **Caudate^b^** | **Anterior Insula**^b^ | **Posterior Insula**^b^ |
| --- | --- | --- | --- | --- | --- | --- |
| **Alcohol** |  |  |  |  |  |  |
| *Number of drinks in preceding week* | 0.64 | 0.71 | 0.28 | 0.15 | 0.30 | 0.66 |
| *Number of drinks in a typical week* | 0.44 | 0.97 | 0.57 | 0.87 | 0.06 | 0.51 |
| **Nicotine** |  |  |  |  |  |  |
| *Age of onset of regular nicotine use* | 0.16 | 0.28 | 0.18 | 0.24 | 0.33 | 0.02 |
| *Current number of daily cigarettes* | 0.99 | 0.90 | 0.24 | 0.78 | 0.74 | 0.54 |
| *Number of days smoking per week* | 0.41 | 0.78 | 0.64 | 0.58 | 0.03 | 0.89 |
| **Marijuana** |  |  |  |  |  |  |
| *Number of lifetime uses* | 0.47 | 0.77 | 0.74 | 0.76 | 0.55 | 0.92 |
| **Impulsiveness (BIS)** |  |  |  |  |  |  |
| *Attention* | 0.80 | 0.57 | 0.68 | 0.71 | 0.69 | 0.49 |
| *Motor* | 0.68 | 0.67 | 0.75 | 0.15 | 0.45 | 0.79 |
| *Cognitive Complexity* | 0.57 | 0.07 | 0.66 | 0.66 | 0.60 | 0.86 |
| **Sensation Seeking (SSS)** |  |  |  |  |  |  |
| *Experience Seeking* | 0.85 | 0.76 | 0.64 | 0.48 | 0.76 | 0.37 |
| *Disinhibition* | 0.23 | 0.80 | 0.64 | 0.38 | 0.96 | 0.46 |
| *Boredom Susceptibility* | 0.75 | 0.86 | 0.51 | 0.63 | 0.41 | 0.49 |

Abbreviations: BIS, Barrett Impulsiveness Scale, SSS, Sensation Seeking Scale, ^a^ refers to whole brain analysis, ^b^ refers to risk mask analysis
